# Cross-Linking Optimization for Electrospun Gelatin: Challenge of Preserving Fiber Topography

**DOI:** 10.3390/polym12112472

**Published:** 2020-10-25

**Authors:** Chiara Emma Campiglio, Selene Ponzini, Paola De Stefano, Giulia Ortoleva, Lorenzo Vignati, Lorenza Draghi

**Affiliations:** 1 Department of Chemistry, Materials and Chemical Engineering “G. Natta”, Politecnico di Milano, via Mancinelli 7, 20131 Milan, Italy; Chiaraemma.campiglio@polimi.it; 2 INSTM—National Interuniversity Consortium of Materials Science and Technology, Local Unit Politecnico di Milano, Piazza Leonardo da Vinci 32, 20133 Milan, Italy; selene.ponzini@mail.polimi.it (S.P.); paola.destefano@polimi.it (P.D.S.); giulia.ortoleva@mail.polimi.it (G.O.); lorenzo4.vignati@mail.polimi.it (L.V.)

**Keywords:** gelatin hydrogels, electrospinning, scaffold, cross-linking, EDC carbodiimide, tissue engineering

## Abstract

Opportunely arranged micro/nano-scaled fibers represent an extremely attractive architecture for tissue engineering, as they offer an intrinsically porous structure, a high available surface, and an ideal microtopography for guiding cell migration. When fibers are made with naturally occurring polymers, matrices that closely mimic the architecture of the native extra-cellular matrix and offer specific chemical cues can be obtained. Along this track, electrospinning of collagen or gelatin is a typical and effective combination to easily prepare fibrous scaffolds with excellent properties in terms of biocompatibility and biomimicry, but an appropriate cross-linking strategy is required. Many common protocols involve the use of swelling solvents and can result in significant impairment of fibrous morphology and porosity. As a consequence, the efforts for processing gelatin into a fiber network can be vain, as a film-like morphology will be eventually presented to cells. However, this appears to be a frequently overlooked aspect. Here, the effect on fiber morphology of common cross-linking protocols was analyzed, and different strategies to improve the final morphology were evaluated (including alternative solvents, cross-linker concentration, mechanical constraint, and evaporation conditions). Finally, an optimized, fiber-preserving protocol based on carbodiimide (EDC) chemistry was defined.

## 1. Introduction

Electrospun fiber substrates have emerged as an excellent platform for tissue engineering (TE) applications as they possess many of the desired properties appropriate for cell and tissue growth, such as an interconnected three-dimensional porous network, a high surface-to-volume ratio and an ideal topography to guide cell migration. Electrospinning is rightly considered a biomimetic process, as the scaffolds produced can closely resemble the micro- and nano-scale architecture of the native extracellular matrix (ECM), that consists of fibrils and fibers with diameters between 50 and 500 nm [1]. The advantages and unique features of electrospun substrates have led to their application in the regeneration of a variety of tissues, including skin, bone, blood vessel, nerve and articular cartilage [2,3,4,5,6]. Naturally occurring biopolymers generally appear as a particularly attractive approach for the fabrication of scaffolds in a variety of hydrogel forms, due to their favorable properties [7,8], but processing into fibers seems an ideal combination of chemicals and architectural features. Accordingly, many biopolymers, polysaccharides and proteins in particular, can be processed into fibers through a variety of techniques [9,10,11], and many of them, but proteins in particular, have been widely processed through electrospinning due to their advantageous biomimicry behavior and intrinsic biocompatibility [12]. Collagenous materials (i.e., collagen and gelatin) in particular appear as the ideal choice, as they constitute the structural support of cells in most tissues. Unfortunately, processed collagen and gelatin are characterized by poor stability and fast degradation and without further treatments they are generally unsuitable for scaffolds fabrication. Similarly, electrospun gelatin and collagen matrices require cross-linking to improve their stability and increase mechanical properties. For the cross-linking of electrospun nanofibers, both physical and chemical methods can be chosen, where the latter generally appear to be more efficient, but also have more potential drawbacks in terms of biocompatibility [13,14]. Physical cross-linking methods mainly include the use of plasmo-chemical treatment [15] or dehydrothermal treatment [16]. They allow for avoiding potentially toxic compounds, which might eventually result in cytotoxic effects, but are generally affected by relatively low efficiency, due to the formation of mainly non-covalent bonds between polymeric chains, and limited stability as a consequence. On the other hand, chemical cross-linking methods involve the formation of covalent bonds between the polymeric chains and result in more stable collagenous scaffolds. For the chemical cross-linking of gelatin and collagen, zero length type cross-linker (where the reagent catalyzes the cross-linking reaction but is eventually removed) and non-zero length type (the cross-linking reagent is incorporated into the polymer network) are used [17]. A resume of the scientific literature presented in the last decade together with the pros and cons of cross-linking strategies typically involved in the stabilization of collagenous electrospun nanofibers can be found in [14].

Among chemical cross-linking strategies, the most widely used zero-length cross-linking method for gelatin electrospun nanofibers relies on the reaction of carbodiimide (i.e., 1-ethyl-3-(3-dimethylaminopropyl)carbodiimide (EDC)) to form amide bonds between polymer chains, while glutaraldehyde (GTA) and genipin are considered the most used non-zero length cross-linkers. Several protocols based on these cross-linkers have been reported for electrospun collagenous substrates, but they involve the use of solvents that dissolve or swell gelatin (water and ethanol in particular). This can result in fused fibers, membrane shrinkage and compromised porosity [18]. Furthermore, while weight loss is frequently analyzed, the effect of water absorption on fibers is barely accounted for. As a consequence, fiber morphology and porosity, already partially compromised by the cross-linking process, are very likely to be almost nullified by swelling in medium. On many occasions, the nanotopography resulting from the electrospinning fabrication process turns into a barely structured film during cross-linking. Unless this issue is accurately addressed, data obtained on the effect of nanofiber topography on cell behavior can hardly be comparable, and in some cases not even completely reliable.

To investigate this specific aspect, and in particular the effect on fiber morphology, before and after water absorption, we applied common protocols for the cross-linking of electrospun gelatin scaffolds. Then, we evaluated the effectiveness of possible strategies to preserve the electrospun morphology with its topographical cues and porosity in turn, as far as possible. More specifically, the effect of different solvents, mechanical constraint during cross-linking, cross-linker concentration, reaction time, and evaporation conditions were investigated in order to stabilize gelatin electrospun substrates for TE applications.

## 2. Materials and Methods 

### 2.1. Materials

All reagents were purchased from Sigma Aldrich (Darmstadt, Germany) and used without further purification, unless otherwise specified. 

### 2.2. Electrospinning of Gelatin Nanofibers 

The substrate for all experiments was prepared by electrospinning Gelatin Type A from porcine skin dissolved at a concentration of 10% *w/v* in a mixture of acetic acid and water (9:1) [19]. The solution was stirred for 1 h at room temperature to obtain complete dissolution. Approximately 5 mL of the polymeric solution was placed in a 20 mL syringe connected to a stainless steel spinneret (ID = 0.030′′) through a polytetrafluoroethylene (PTFE) 16 Gauge capillary tube. The syringe was loaded in a KD Scientific syringe pump (Model 100, Holliston, MA, USA) to control the flow rate at 0.5 mL/h. The solution was spun towards a rectangular aluminum plate for creating randomly oriented, non-woven, fibrous matrices. The spinneret was placed 13 cm from the collector and a 15 kV voltage supply was used to charge the solution and the collector. The electrospinning process was performed at room temperature and with a relative humidity in the range of 20–40%.

### 2.3. Analysis of Fiber Morphology in Replicated Literature Protocols

Different chemical cross-linking protocols (i.e., EDC, genipin, vapor of GTA) with parameters derived from scientific literature were firstly applied [16,20,21], to evaluate the morphology obtained and to serve as a reference for further investigation on the parameters that could affect nanofibers morphology during the cross-linking process.

### 2.4. Definition of New Protocols: Analysis of Factors Involved in Fiber Fusion

#### 2.4.1. Selection of Non-Swelling Solvents

In the search for a solvent with possibly negligible influence on fiber morphology, the effect of different polar (ethanol, acetonitrile, ethyl acetate, tert-butanol) and non-polar (acetone, chloroform, diethyl ether, hexane) solvents on fibers morphology was qualitatively evaluated by scanning electron microscope (SEM, Stereoscan 360 Cambridge instruments, London, UK) after having left electrospun gelatin matrices immersed in each solvent for 24 h and dried.

#### 2.4.2. EDC/NHS Cross-Linking in Non-Swelling Solvents

After solvents with a negligible effect on fibers morphology were identified, the possibility to use them to dissolve 1-ethyl-3-(3-dimethylaminopropyl)carbodiimide (EDC) for cross-linking electrospun gelatin was evaluated. To optimize the process, different molarities of cross-linker (25–50–75 mM) and reaction times (2, 8, 18 h) were evaluated. According to the scientific literature, N-hydroxysuccinimide (NHS) was included in the EDC coupling protocol to improve reaction efficiency [16]. After cross-linking, electrospun mats were washed in the same solvent used for reaction and air-dried (Supplementary information: Figure S1). 

To evaluate the effect of water on the fiber morphology and preliminarily assess the effectiveness of cross-linking, mats were immersed in distilled water for 10 min. 

#### 2.4.3. Glutaraldehyde Cross-Linking in Non-Swelling Solvent

With the aim to always obtain a greater control over the cross-linking reaction, glutaraldehyde (GTA) cross-linking in non-swelling solvent was evaluated as an alternative to its vapor. GTA was dissolved in tert-butanol (t-BuOH) for the cross-linking of gelatin nanofibers according to Skotak et al.’s protocol [22]. Different concentrations of GTA (0.5–1–2%) and reaction times (30 min, 1 h, 2 h) were investigated.

#### 2.4.4. Mechanical Constraint 

**Figure 1:**
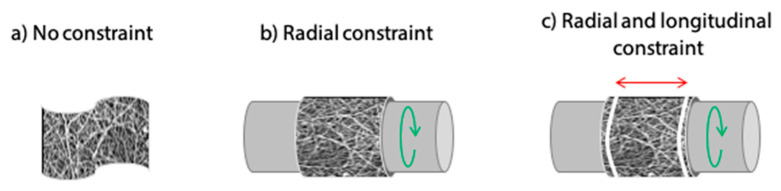
Schematic representation of electrospun matrix supports for cross-linking. (**a**) No-constraint with sample free-flowing in the solution. (**b**) Radial constraint on a mandrel. (**c**) Gelatin matrix undergoes radial and longitudinal constraint imposed by tubular support and block rings, respectively.

As the proximity of fibers during the process increases the possibility of inter-fiber cross-linking, mechanical constraint was applied to the electrospun matrices during cross-linking to keep fibers separated and limit their fusion. Three different conditions were compared: no constraint (i.e., samples free-flowing in the solution), radial constraint, and both radial–longitudinal constraint (Figure 1).

#### 2.4.5. Effect of Solvent Evaporation

As solvent evaporation can also involve damage to fiber structure as a consequence of surface tension action, different drying conditions after cross-linking were evaluated. More specifically, three different conditions were compared: simple air drying, slow drying in a closed vessel and slow-drying in a closed vessel with silica gel beads inserted to absorb air humidity (Supplementary information: Figure S1). 

### 2.5. Characterization of Cross-Linked Gelatin Nanofibers 

#### 2.5.1. Morphological Analysis

The morphology of electrospun gelatin matrices was investigated by scanning electron microscope (SEM, Stereoscan 360 Cambridge instruments, London, UK) to evaluate the influence of processing steps on fiber morphology. All samples were sputter-coated with gold and observed using an accelerating voltage of 10 kV.

#### 2.5.2. Weight Loss and Stability

Swelling and weight loss of cross-linked gelatin matrices was assessed at 37 °C in phosphate buffer saline (PBS). Briefly, cross-linked samples (*n* = 3 per type, 5 mm × 5 mm) were placed in 24-multiwell, covered with 1 mL of PBS sealed and stored at 37 °C. Sodium azide (0.02% *w/v*) was added as a biocide agent to prevent contamination. At set time points (1 h, 48 h and 7 days), samples were removed from the solution, gently swabbed with tissue paper to remove the excess of water and frozen at −20 °C. Samples were then lyophilized and weighted in dry state with precision balance. The weight variation (Δ*W*%) was calculated as:

(1) $$∆W\%=\frac{W_{t}-W_{0}}{W_{0}}\;100$$

were *W*_t_ is the weight of the freeze-dried sample at time point t and *W*_0_ is the initial dry weight of the sample.

#### 2.5.3. Evaluation of Cross-Linking Degree

The determination of cross-linking degree was carried out using a TNBS (2,4,6-Trinitrobenzene Sulfonic Acid) method, with some modifications with respect to [16]. The purpose of this assay was to quantify the variation in free amine groups of gelatin before and after cross-linking and the number of reacted groups as their difference. TNBS test was performed on 3 samples for each cross-linking method (i.e., EDC/NHS, GTA solution) using non cross-linking gelatin as control. In addition, for EDC/NHS cross-linking method an investigation on the effect of reaction time on cross-linking degree was carried out. 

Briefly, gelatin samples were weighted in a test tubes where 1 mL of 0.5% *w/v* TNBS solution and 1 mL of 0.1 M sodium hydrogen carbonate (NaHCO_3_, pH 8.5) were added. After heating at 40 °C for 2 h, each sample was further treated with 2 mL of 6 M HCl at 60 °C for 1.5 h. The absorbance of the resulting solutions was determined by a spectrophotometer (Tecan, Genios Plus plate reader, Mannedorf, Switzerland) at 360 nm. 

The degree of cross-linking (*CD*%) was then calculated using the following equation:

(2) $$CD\;\%=\left(1-\frac{A_{c}}{A_{nc}}\right)\;100$$

where *A_c_* and *A_nc_* represent the absorbance of the cross-linked and pristine gelatin samples, respectively.

#### 2.5.4. Mechanical Characterization

The mechanical properties of the EDC/NHS cross-linked (wet and dry condition) and non-cross-linked (dry condition) gelatin nanofibers samples were tested using a Dynamic Mechanical Analyser (DMA Q800, TA Instruments, New Castle, DE, USA). Uniaxial tensile testing was performed in triplicate on rectangular shaped samples for each type (length = 25 mm, width = 5 mm), applying a preloaded force of 0.005 N and a ramp force of 0.05 N/min. 

#### 2.5.5. In Vitro Biological Characterization: Cytotoxicity Test on Extracts

For cytotoxicity assessment on cross-linked matrices, samples eluates were obtained, according to UNI EN ISO 10993-5, by incubating the samples (i.e., EDC/NHS, and GTA solution cross-linked matrices) in culture medium for 1 h and 24 h. The medium was composed by Dulbecco’s Modified Eagle Medium (DMEM), Fetal Bovine Serum (FBS) 10% *v/v*, penicillin/streptomycin 1% *v/v*, glutamine 2 mM, Hepes 10 mM and sodium pyruvate 1 mM. 

HeLa cells (epithelial cell line from cervix carcinoma) were seeded at a density of 10^4^ cells/well in 96-wells plates and cultured with fresh complete medium until 70% confluent. The medium was then replaced with eluates or control (i.e., medium aged for the same time) and cells were returned to the incubator. After 24 h, cell metabolic activity was assessed by Alamar Blue™ assay, according to manufacturer recommendations, and the fluorescence of each sample was spectrophotometrically read in a multiwell plate reader (Tecan, Genios Plus, Mannedorf, Switzerland).

### 2.6. Statistical Analysis

Data are expressed as mean ± standard deviation (SD). Significant differences between two sets of data were determined by One-way ANOVA followed by Tukey’s post-hoc test for pairwise comparisons. *p* < 0.05 was considered statistically significant (* *p* < 0.05, ** *p* < 0.01, *** *p* < 0.001). 

## 3. Results

**Figure 2:**
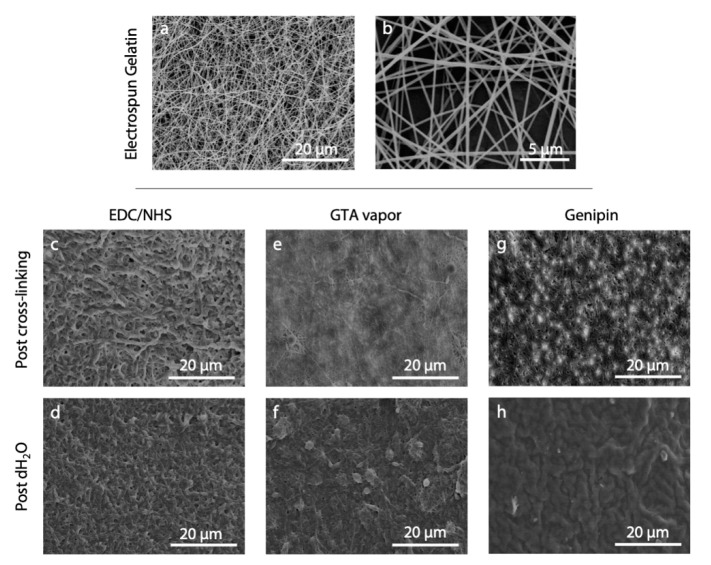
(**a**,**b**) SEM micrographs of electrospun gelatin nanofibers as appeared after the electrospinning process. (**c**–**h**) Results obtained from the application of different cross-linking protocols: (**c**,**d**) EDC/NHS [20], (**e**,**f**) GTA vapor [16], and (**g**,**h**) genipin [21]. SEM micrographs show nanofiber morphology after the cross-linking process (**c**,**e**,**g**) and after the immersion of the cross-linked matrices in distilled water (**d**,**f**,**h**). Scale bars represent 20 μm (**a**,**c**–**h**) and 5 μm (**b**).

Electrospinning of gelatin Type A from acetic acid/water solution (9:1) resulted a continuous and stable process. It allowed for the formation of homogeneous non-woven matrices made up of nanofibers that displayed a random orientation and an average diameter of 268.3 ± 17.7 nm (Figure 2a,b).

The morphology obtained by applying the three selected literature protocols (i.e., EDC/NHS, GTA vapor, and genipin) is shown in Figure 2c–h. As can be observed, the protocols were not effective under our conditions and we were unable to obtain well-preserved fiber morphology, either after the cross-linking nor after the immersion of the matrices in water. SEM images showed swelled fibers after cross-linking, and they were barely recognizable after immersion in water.

Among the most widely used strategies proposed for the stabilization of collagenous materials (i.e., EDC/NHS, GTA vapor, GTA solution, genipin and others [14]), the use of cross-linking solutions has been proved to be more effective than the employment of vapors, light and heat to stabilize nanofibers substrates [16,23]. Thus, EDC/NHS and GTA in solution were selected for further analysis in order to evaluate the effect of cross-linking parameters on fibers morphology.

### 3.1. Selection of Non-Swelling Solvents

**Figure 3:**
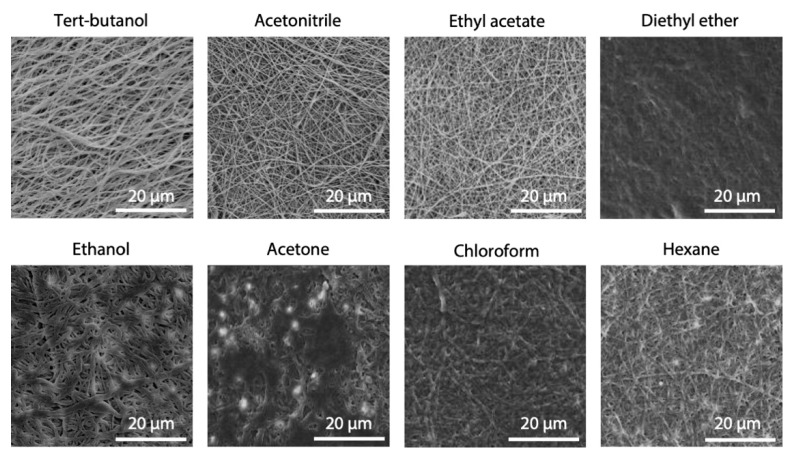
SEM images of electrospun gelatin fibers after 24 h of immersion in different solvents. Scale bars represent 20 μm.

The morphology of electrospun fibers after 24 h of immersion in different solvents, selected as possible medium candidates for cross-linking, are shown in Figure 3. Acetonitrile, ethyl acetate and tert-butanol, in particular, were the most effective in preserving nanofibers morphology and were selected for further testing. On the contrary, diethyl ether, chloroform and acetone caused a consistent swelling of fibers, but ethanol and hexane also had the same effect, although to a lower extent.

### 3.2. EDC/NHS Cross-Linking Protocol Optimization in Non-Swelling Solvents

All EDC/NHS protocols proposed in the literature involve the use of ethanol or water for the dissolution of carbodiimide. However, as ethanol itself was found to induce appreciable swelling of fibers (Figure 3), acetonitrile and ethyl acetate were evaluated as possible alternatives. 

EDC molarity was used in large excess and varied from 25 to 75 mM and was added to the reaction mixture keeping the EDC/NHS ratio to 2.5:1 [16]. EDC activates the carboxyl groups of gelatin, which can react with free amino groups, hydrolyze or rearrange to O-acylisourea residues. By adding NHS, the efficacy of the reaction usually increases, because NHS reacts with the activated groups forming an intermediate that is less susceptible to hydrolysis and rearrangement of the carboxylic acid groups [24]. Together with the influence of EDC concentration, the reaction time was evaluated, leaving electrospun gelatin matrices in the cross-linking solution for 2, 8 and 18 h. 

Results obtained showed that the cross-linking reaction was also possible with the selected alternative solvents, and stability in water of electrospun gelatin was obtained.

**Figure 4:**
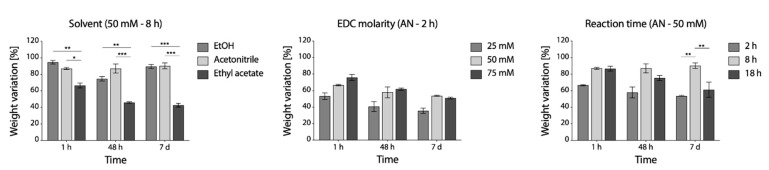
Percentage weight variation of EDC/NHS cross-linking samples in PBS at 37 °C up to 1 week. Left to right: effect of solvent, effect of EDC molarity and effect of reaction time (* *p* < 0.05, ** *p* < 0.01, *** *p* < 0.001).

From weight loss evaluation (Figure 4), acetonitrile was appeared as the most effective alternative. Compared to ethanol, used as the control, its effect on the stability of fibers was similar. Contrarily, when ethyl acetate was used, a consistent sample weight loss over time was observed (about 60% after 7 days). In acetonitrile, EDC molarity of 50 mM appeared as reasonable compromise between effectiveness and reagent consumption, although differences were not significant, while 8 h was found as the more appropriate reaction time.

The use of acetonitrile (i.e., polar aprotic solvent), together with the optimization of the reaction time (8 h) and of the EDC molarity (50 mM, NHS molarity is subsequently 20 mM according to an EDC/NHS ratio of 2.5:1) allowed to obtain an effective cross-linking of gelatin nanofibers.

### 3.3. GTA Cross-Linking Protocol Optimization in Non-Swelling Solvent

**Figure 5:**
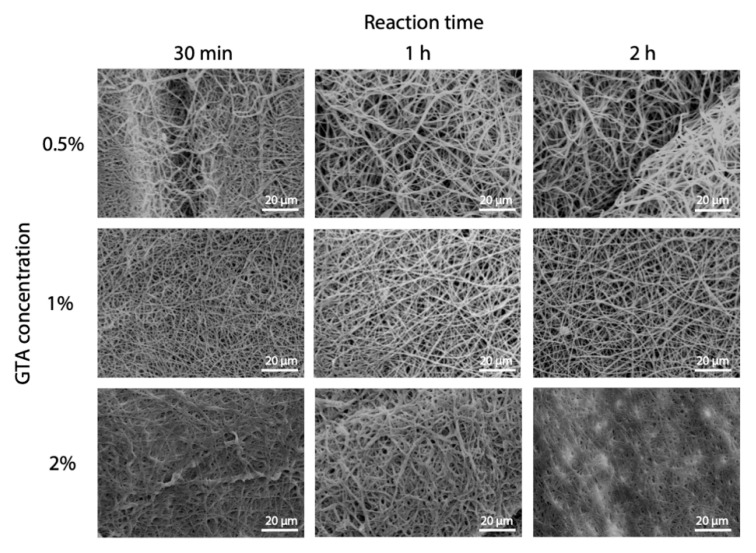
GTA cross-linking parameters optimization. SEM micrographs of electrospun gelatin cross-linked in GTA solution showing the effect of GTA concentration and reaction time. Scale bars represent 20 μm.

Optimization of cross-linking protocol was also performed for GTA in solution. In this case, tert-butanol was used as it has previously been shown as an effective solvent for GTA [22]. Working in solution instead of vapor, a more common method was aimed at having more control on the process. As for EDC, different concentrations and reaction times were tested. The images of fibers acquired by SEM are shown in Figure 5, and it can be observed that a good fibers morphology is preserved for lower concentrations. On the contrary, higher GTA concentration (2%) determined a consistent swelling of fibers both at low and high cross-linking reaction times.

**Figure 6:**
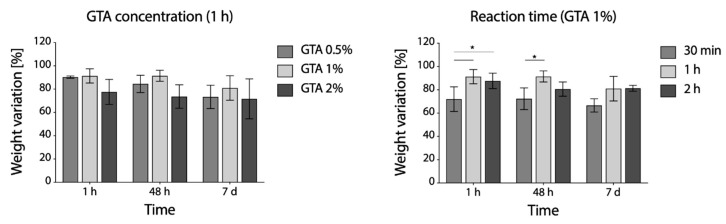
**Percentage** weight variation of GTA cross-linked samples immersed in PBS at 37 °C: effects of GTA concentration (**left** panel) and reaction time (**right** panel) on weight loss evaluated over time (* *p* < 0.05).

According to weight loss variation, increasing GTA concentration over 1% not only did not improve the outcome of cross-linking, but even seemed to be detrimental (Figure 6, left panel) and 1 h was shown to be the more effective reaction time (Figure 6, right panel).

### 3.4. Evaluation of Cross-Linking Degree

To confirm the successful cross-linking for the two developed protocols, the reduction in the free amino group content was evaluated by TNBS assay. As reasonably expected, the GTA cross-linking method was shown to be extremely effective, and a cross-linking degree above 99% was calculated.

**Figure 7:**
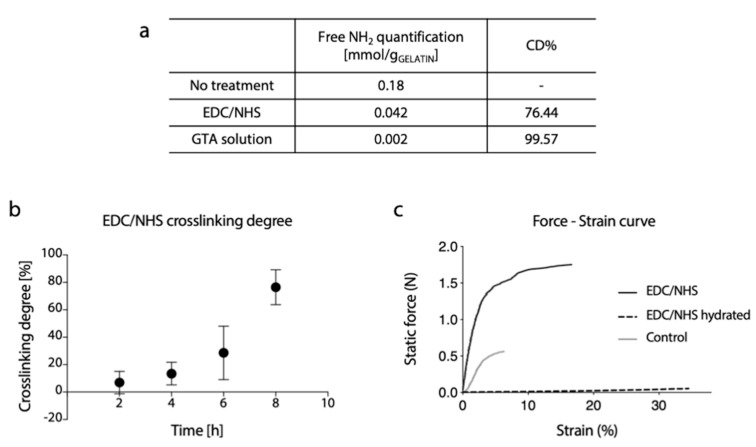
(**a**) Free amino group quantification and related percentage of cross-linking degree of gelatin electrospun matrices cross-linked by EDC/NHS or GTA solution method. (**b**) Percentage of cross-linking degree at different reaction times of EDC/NHS cross-linked matrices. (**c**) Representative force-strain curves of gelatin electrospun matrices cross-linked with EDC/NHS (dry and hydrated state) and non-cross-linked (control, dry state).

For EDC/NHS, on the other hand, the increase in cross-linking degree was exponentially proportional to the reaction time, but a lower cross-linking degree (76%) was obtained for the higher reaction time (Figure 7b).

Representative curves of the mechanical response of cross-linked (dry and hydrated conditions) and un-crosslinked samples during the tensile test are shown in Figure 7c. As it can be observed, the mechanical properties of dry cross-linked gelatin nanofibers membrane were substantially increased. As reasonably expected, a marked reduction in stiffness and increase in elongation at break were observed in the hydrated state. 

### 3.5. Cross-Linking Set-Up: Effect on Nanofibers Morphology

**Figure 8:**
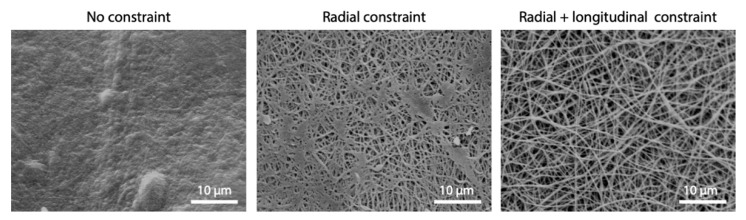
Effect of mechanical constraint imposed during EDC/NHS cross-linking process on fibers morphology (cross-linking parameters: acetonitrile, 50 mM EDC, 8 h). Scale bars represent 10 μm.

Constraining electrospun matrices during cross-linking was also found as an effective method to better preserve the fibrous structure. As it can be observed in Figure 8, improvements were obtained with radial constraint only, while they were even more considerable when longitudinal constraint was added to prevent shrinkage.

### 3.6. Controlled Solvent Evaporation after Cross-Linking Process

**Figure 9:**
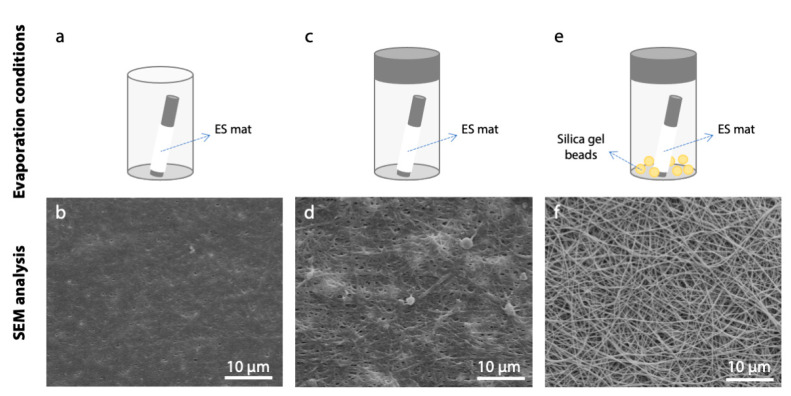
Effect of different evaporation conditions on fiber morphology after cross-linking process (acetonitrile, 50 mM EDC, 8 h). In the lower row, SEM micrographs of gelatin nanofibers are reported (scale bars represent 10 μm). (**a**,**b**) Air drying, (**c**,**d**) evaporation in a closed vessel, and (**e**,**f**) evaporation in a closed vessel containing silica gel beads.

Despite the parameter optimization performed, poor results in terms of fibers morphology maintenance were still obtained. To overcome this limitation, an additional variable was taken into consideration and a study on the effect of different evaporation conditions was performed. Together with the constraint applied on matrices, the evaporation conditions had a great effect on the preservation of nanofibers morphology. As it was demonstrated by SEM analysis (Figure 9), the evaporation of cross-linking solvents must be performed in a controlled environment and with a low rate. The presence of silica gel beads in the container resulted a crucial parameter during this process. Silica gel beads were used as a desiccant to reduce humidity in the closed container during solvent evaporation, as this resulted in better preservation of nanofibrous structure. 

### 3.7. In vitro Biological Characterization

**Figure 10:**
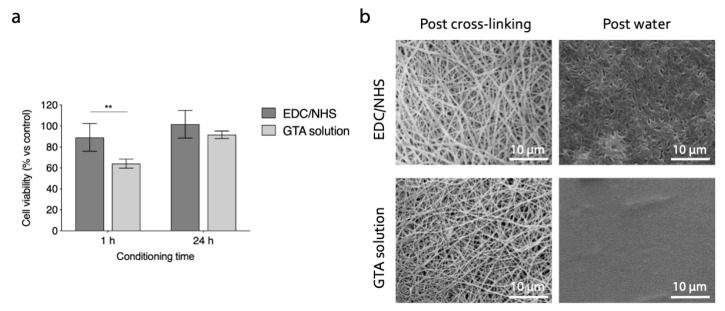
(**a**) In vitro indirect cytotoxicity of cross-linked gelatin nanofibers. Cell viability (% vs. control) of HeLa cells cultured in EDC/NHS and GTA samples eluates (** *p* < 0.01). (**b**) SEM micrographs of electrospun gelatin cross-linked with optimized protocols. Fibers morphology is shown after the cross-linking process and after the immersion of matrices in distilled water (10 min). Scale bars represent 10 μm.

The cytotoxicity of the electrospun gelatin matrices, cross-linked with the two optimized methods (i.e., EDC/NHS, GTA solution) was evaluated in vitro by culturing HeLa cells in culture medium eluates, obtained by placing in contact the culture medium with gelatin matrices specimens for 1 and 24 h. The viability of cells cultured in contact with eluates is shown in Figure 10a, and was significantly higher for samples cross-linked with carbodiimide. The GTA eluates became cytotoxic after 1h, confirming the results presented in several scientific works regarding the possible cytotoxic effects of this cross-linker [25,26]. However, the results after 24 h were statistically not different, to suggest that long preconditioning of samples can be an effective strategy to avoid the cytotoxicity in GTA cross-linked samples.

The final results obtained with the cross-linking methods considered are showed in Figure 10b. Both the strategies involved (i.e, EDC/NHS: 50 mM, 8 h, acetonitrile; GTA solution: 1% *v/v*, 1 h, t-BuOH), with the cross-linking set-up optimized, allow us to obtain morphologically stable fibrous networks. In particular, the nanofiber morphology was well preserved after the cross-linking process. However, the immersion of matrices in aqueous environment still caused a partial swelling of fibers. In the samples cross-linked with EDC/NHS the nanofibrous topography was preserved even after the immersion in distilled water. On the contrary, GTA solution cross-linking did not allow us to obtain a suitable stabilization of nanofibrous network, and resulted in a film-like surface after the immersion in an aqueous environment. This morphological characterization pointed out that the carbodiimide cross-linking protocol optimized in this study was promising for the stabilization of electrospun gelatin nanofibrous substrates, aiming at their application as cells scaffolds.

## 4. Discussion

Collagenous fibrous architectures are exceptional substrates for cell growth, as they can actually mimic the native extracellular matrix and offer to cells a variety of stimuli to guide their behavior. On the other hand, electrospinning of gelatin and collagen is a simple and effective technique for preparing sub-micron fibrous structures. As a consequence, electrospun gelatin and collagen mats are extremely popular materials in tissue engineering and regenerative medicine [3,27,28].

Many studies have focused on demonstrating the effectiveness of contact guidance of sub-micrometric fibers features [29,30,31]. However, preserving the as-spun fibrous structure and scaffold porosity is should not be taken for granted for hydrogel materials. Synthetic polymers, such as PCL, to cite a popular material for electrospinning, do not require cross-linking and have negligible water absorption. As a consequence, they give guarantees that fibrous structure and porosity will be well preserved during cell culture. This is not necessarily true for natural hydrogel forming materials. Fiber swelling and fusion during cross-linking and the consistent water absorption can easily cause the electrospinning membrane to turn into a compact film. Hence, the control on structure in every step of the process is crucial and the optimization of cross-linking protocol that allows the stabilization of the scaffold while preserving the ECM-like architecture obtained during the fabrication process is essential. For this reason, we have decided to investigate the effect of different cross-linking parameters on the morphology of electrospun gelatin fibers, with the final aim to develop an effective protocol and ensure that a proper microstructure is eventually offered to cells.

The choice of a suitable cross-linking agent requires knowledge of the reactive groups present, and of the appropriate ambient conditions (e.g., solvent, reaction time and temperature) that do not negatively affect the protein. Compared to their physical counterpart, chemical cross-linking methods are more effective due to their ability to form covalent bonds between gelatin polymer chains, allowing for the formation of a stable cross-linked network. They were employed for the stabilization of different types of gelatin scaffolds, such as hydrogels [32], porous scaffolds [33], films [34] and ultrafine fibers [35]. Among chemical cross-linking methods used for gelatin nanofibers, heterobifunctional carbodiimides, and in particular EDC, is of great interest in maximizing the extent of cross-linking, because EDC molecules contain two different reactive groups that are able to directly link two different amino acid side chains [36]. Moreover, EDC, used with or without NHS, is able to introduce a stable chemical cross-link between gelatin molecules without introducing any spacers or external sequence (i.e., a zero length cross-linker). 

Several studies investigate the ability of carbodiimide in stabilizing collagenous materials [20,35,37,38,39]. However, the majority of them use ethanol as solvent for the dissolution of EDC, but modified morphology are sometimes reported and we were unable to find data or results to support the preservation of fibrous morphology after immersion in an aqueous environment for pure gelatin (blends with PCL are much more common [40]). As an impairment to the fibrous network can be caused by the solvent itself, we have decided to propose the use of acetonitrile instead of ethanol to dissolve the cross-linker. This choice was made after testing different solvents and verifying that ethanol induces a consistent swelling of gelatin fibers (Figure 3). A possible explanation of this effect resides in the characteristics of these solvents. Ethanol and acetonitrile are both polar solvents, but they differ in protic/aprotic behavior. Ethanol (dielectric constant = 25) is a polar protic solvent that can participate in the formation of hydrogen bonding due to the presence of –OH groups, while acetonitrile (dielectric constant = 37), that is a polar aprotic solvent, cannot form this type of bond. This means that ethanol can induce fiber swelling due to its ability to form hydrogen bonding within gelatin polymer chains. In addition, its protic nature can hinder the interaction between the protonated carbodiimide and the carboxylic functional groups of aspartic and glutamic amino acids of gelatin, acting as a competitor during the cross-linking reaction. The use of acetonitrile as a cross-linking solvent, together with optimized parameters (EDC molarity and reaction time), allowed us to induce a good stability in the nanofibrous matrices, confirming the efficacy of the carbodiimide in stabilizing collagenous materials. At this point, the critical issue involved was not to provide better reactivity of the groups but to provide the conditions that maintained the proper structure of the fibrous matrix. Concerning this issue, specific attention was given to the cross-linking set-up during and after the process. Promising results were obtained, preventing the shrinkage of fibrous matrices during the cross-linking. The application of a radial-longitudinal constraint to the gelatin fibers allowed a homogeneous infiltration of the cross-linker, promoting the formation of amide bonds within the single fiber and not between neighboring fibers. Moreover, a slow and controlled evaporation of solvent after the process allowed us to perfectly preserve the nanofibrous morphology of electrospun matrices (Figure 9f). 

The cross-linking strategy allowed us to preserve the nanofiber architecture created by electrospinning, avoiding any undesirable swelling of fibers. Despite this promising outcome, the morphological appearance of nanofibers after the immersion in an aqueous environment (Figure 10b) undergo a change: a reduced porosity was observed together with partial fiber swelling. The topographical cues offered by a nanostructure surface are still presented and can offer a suitable substrate for cell adhesion and proliferation [41]. Furthermore, the nanoporosity of the network can guarantee adequate exchange of nutrients and waste products, essential characteristics for the development of a tissue engineering scaffold. Cross-linked gelatin matrices were also stable in aqueous solution, showing a weight loss of 10 ± 3% after 7 days (Figure 4). The result proved the efficacy of the cross-linking treatment, aiming at the stabilization of gelatin nanofibers in a physiological-like environment. 

In order to evaluate the degree of the cross-linking reached with the proposed EDC/NHS protocol, the TNBS biochemical assay was satisfactory in measuring the percentage of free amino groups. The results reveal that the use of carbodiimide is an effective strategy to stabilize gelatin nanofibers, forming amide bonds between carboxylic groups and amine groups of polymer chains. Moreover, the performed assay highlighted the relationship between the reaction time and the percentage cross-linking degree that exists when EDC/NHS is used as cross-linker.

Finally, the non-cytotoxicity of EDC/NHS cross-linked sample was proven, confirming that, after a possible in vivo application, no cytotoxic effects would be observed on the cell components that allow for tissue regeneration. Moreover, this result proved the superiority of the carbodiimide cross-linking strategy over the widely used glutaraldehyde method. In agreement with other works [42,43], the indirect cytotoxicity outcomes confirmed that GTA, without proper preconditioning, can have negative effects on cells viability. 

All the above-mentioned reasons have proven the efficacy of the cross-linking strategy here developed. The use of EDC/NHS cross-linker allows for the stabilization of electrospun gelatin nanofibers, and more importantly, allows us to preserve the topographical cues offered by an electrospun substrate, a crucial feature for the fabrication of adequate scaffolds for tissue engineering.

## 5. Conclusions

The major concerns in developing a protocol for the cross-linking of electrospun gelatin substrates for use in tissue engineering applications are maintaining the nanofibrous structure, preventing any cytotoxic effects and imparting desirable mechanical properties. Gelatin is a widely available biopolymer and electrospun gelatin scaffolds can closely mimic the biochemical and ultrastructural properties of the native ECM of tissues, which is well known to influence cell behavior. The carbodiimide cross-linking protocol we developed, fulfills all the above-mentioned aims. The use of EDC/NHS in acetonitrile allows us to obtain good stability in a physiological-like environment. Although these are not generally considered as relevant factors, the introduction of a mechanical constraint on fibers and a controlled evaporation condition during and after the cross-linking process, were crucial for fiber morphology preservation. 

Based on the obtained results, a higher attention on the abovementioned aspects analyzed can significantly improve the morphology of cross-linked structures and better support the results of studies focusing on the effect of microstructure on cell response.

## Supplementary Materials

The following are available online at https://www.mdpi.com/2073-4360/12/11/2472/s1, Figure S1: Cross-linking process for EDC/NHS and GTA solution method. Gelatin samples were mounted on a mandrel and immersed in cross-linking solutions for the selected time. After cross-linking, solvent evaporation was carried out under three different conditions: air drying, slow drying in a closed vessel and slow drying in a closed vessel with silica gel beads, in order to select the most suitable condition for fiber topography preservation.
