# Supplementary material for: Cross-Linking Optimization for Electrospun Gelatin: Challenge of Preserving Fiber Topography
Source: Polymers (Basel). 2020 Oct 25;12(11):2472. doi: 10.3390/polym12112472 (PMC7692762; doi:10.3390/polym12112472)
Supplement: Supplementary file 1 [file polymers-12-02472-s001.pdf]

Supplementary Information

Cross-Linking Optimization for Electrospun Gelatin: Challenge of Preserving Fiber Topography

Chiara Emma Campiglio [1], Selene Ponzini [2], Paola De Stefano [2], Giulia Ortoleva [2], Lorenzo Vignati [2] and Lorenza Draghi [1,2,*]

[1] Department of Chemistry, Materials and Chemical Engineering "G. Natta", Politecnico di Milano, via Mancinelli 7, 20131 Milan, Italy; Chiaraemma.campiglio@polimi.it

[2] INSTM—National Interuniversity Consortium of Materials Science and Technology, Local Unit Politecnico di Milano, Piazza Leonardo da Vinci 32, 20133 Milan, Italy; selene.ponzini@mail.polimi.it (S.P.); paola.destefano@polimi.it (P.D.S.); giulia.ortoleva@mail.polimi.it (G.O.); lorenzo4.vignati@mail.polimi.it (L.V.)

* Correspondence: lorenza.draghi@polimi.it; Tel.: +39-0223993184

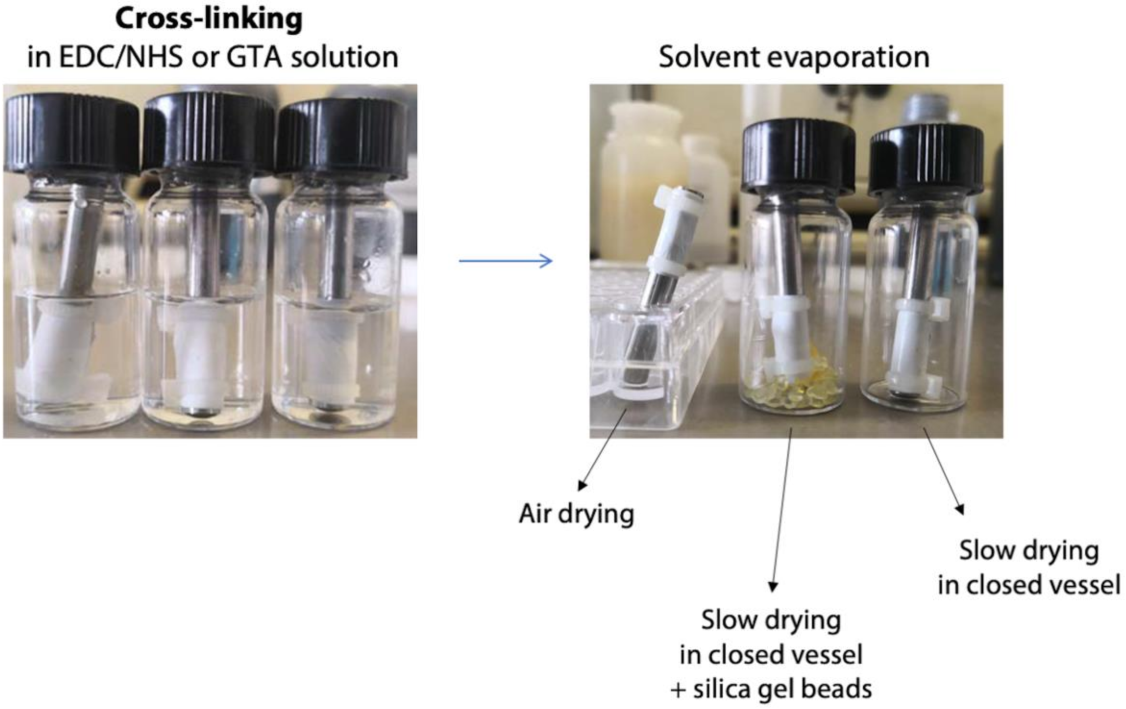

Figure S1. Cross-linking process for EDC/NHS and GTA solution method. Gelatin samples were mounted on a mandrel and immersed in cross-linking solutions for the selected time. After cross-linking, solvent evaporation was carried out under three different conditions: air drying, slow drying in a closed vessel and slow drying in a closed vessel with silica gel beads, in order to select the most suitable condition for fiber topography preservation. .
